# Importance of Cellular Immunity and IFN-γ Concentration in Preventing SARS-CoV-2 Infection and Reinfection: A Cohort Study

**DOI:** 10.3390/v15030792

**Published:** 2023-03-20

**Authors:** Dragan Primorac, Petar Brlek, Eduard Stjepan Pavelić, Jana Mešić, David Glavaš Weinberger, Vid Matišić, Vilim Molnar, Saša Srića, Renata Zadro

**Affiliations:** 1 St. Catherine Specialty Hospital, 10000 Zagreb, Croatia; 2 School of Medicine, Josip Juraj Strossmayer University of Osijek, 31000 Osijek, Croatia; 3 Medical School, University of Split, 21000 Split, Croatia; 4 Department of Biochemistry & Molecular Biology, The Pennsylvania State University, State College, PA 16802, USA; 5 The Henry C. Lee College of Criminal Justice and Forensic Sciences, University of New Haven, West Haven, CT 06516, USA; 6 Medical School REGIOMED, 96450 Coburg, Germany; 7 Medical School, University of Rijeka, 51000 Rijeka, Croatia; 8 Faculty of Dental Medicine and Health, Josip Juraj Strossmayer University of Osijek, 31000 Osijek, Croatia; 9 Medical School, University of Mostar, 88000 Mostar, Bosnia and Herzegovina; 10 National Forensic Sciences University, Gujarat 382007, India; 11 University Hospital Centre Zagreb, 10000 Zagreb, Croatia

**Keywords:** COVID-19, SARS-CoV-2, cellular immunity, Omicron variant, infection, vaccination, humoral immunity

## Abstract

Recent studies have highlighted the underestimated importance of the cellular immune response after the emergence of variants of concern (VOCs) of SARS-CoV-2, and the significantly reduced neutralizing power of antibody titers in individuals with previous SARS-CoV-2 infection or vaccination. Our study included 303 participants who were tested at St. Catherine Specialty Hospital using the Quan-T-Cell SARS-CoV-2 in combination with the Quan-T-Cell ELISA (Euroimmun Medizinische Labordiagnostika, Lübeck, Germany) for the analysis of IFN-γ concentration, and with Anti-SARS-CoV-2 QuantiVac ELISA IgG (Euroimmun Medizinische Labordiagnostika, Lübeck, Germany) for the detection of human antibodies of the immunoglobulin class IgG against the S1 domain of the SARS-CoV-2 spike protein. The statistical analysis showed a significant difference in the concentration of IFN-γ between reinfected participants and those without infection (*p* = 0.012). Participants who were not infected or reinfected with SARS-CoV-2 after vaccination and/or previous SARS-CoV-2 infection had a significantly higher level of cellular immunity. Furthermore, in individuals without additional vaccination, those who experienced infection/reinfection had significantly lower levels of IFN-γ compared to uninfected participants (*p* = 0.016). Our findings suggest a long-lasting effect of cellular immunity, measured by IFN-γ concentrations, which plays a key role in preventing infections and reinfections after the emergence of SARS-CoV-2 variants of concern.

## 1. Introduction

Infection with the SARS-CoV-2 virus leads to pathophysiological mechanisms involving a wide range of biomolecules, such as IFN-γ, that have a protective role during the early stages of the disease [1]. The secretion of IFN-γ activates M1 macrophages and increases macrophage expression of MHC antigens, which facilitates antigen presentation to T cells and therefore plays an important role in the long-lasting cellular immune response [2,3,4,5]. Recent studies have shown the underestimated importance of the cellular immune response after the emergence of the highly infectious Omicron variant of SARS-CoV-2 and the significantly reduced neutralizing power of antibody titers in individuals with previous SARS-CoV-2 infection or vaccination [6,7]. Research on macaques suggests that CD8+ T cells can continue to be protective when neutralizing antibody titers decline or are below the threshold of host protection [8,9]. However, the importance of the level of cellular immunity that would protect against SARS-CoV-2 reinfection is still not determined in humans, and a crucial problem is the lack of data on the protection level of cellular immunity in a larger cohort [10,11,12,13].

Our study aimed to determine the importance of the cellular immune response and its role in preventing infection and/or reinfection in 303 study participants up to 12 months after measuring the concentrations of IFN-γ, a protein produced most abundantly by NK cells, type 1 CD4+, CD8+, and gamma delta (γδ) T cells. IFN-γ has numerous roles in cellular immunity crucial for protection against SARS-CoV-2 infection, including the direct killing of the virus, activation and induction of M1 macrophage production, production of reactive oxygen and nitrogen intermediates, and increased macrophage expression of MHC antigens, which facilitates antigen presentation to T lymphocytes [14]. Therefore, in this study, by measuring the concentration of IFN-γ, we showed differences in the risk of reinfection with SARS-CoV-2.

The aim of our study was to investigate whether the level of cellular immunity has an impact on COVID-19 infection and/or re-infection. Moreover, the purpose was to elucidate whether individuals who were not infected or reinfected with the SARS-CoV-2 virus after vaccination and/or previous COVID-19 infection have a higher level of cellular immunity as measured by the concentration of IFN-γ compared to the antibody concentration of the immunoglobulin class IgG against the S1 domain of the SARS-CoV-2 spike protein. 

## 2. Materials and Methods

### 2.1. Participants, Data Collection, and Retrospective Analysis

The study included 303 participants who were tested for cellular and humoral immunity at St. Catherine Specialty Hospital. All participants filled out a detailed questionnaire on previous SARS-CoV-2 infection and/or vaccination. At the beginning of the study, we took blood from the participants and measured IFN-γ concentration. Participants were monitored for a period of up to 12 months. During that period, we collected data on whether participants were infected, reinfected, or additionally vaccinated and retrospectively associated their IFN-γ concentration measured at the beginning of the study. 

### 2.2. Cellular Immunity Analysis

As described in our previous study, for the analysis of cellular immunity, the Quan-T-Cell SARS-CoV-2 in combination with the Quan-T-Cell ELISA (Euroimmun Medizinische Labordiagnostika, Lübeck, Germany) was used [15]. The principle of the test is a measurement of IFN-γ concentration released by activated immune cells. Fresh whole blood samples were collected in heparinized tubes and pipetted into the three stimulation tubes (Quan-T-Cell SARS-CoV-2): (1) COV-2 IGRA (interferon-gamma release assay) Blank was used for measuring individual IFN-γ concentrations as it contained no activating components; (2) CoV-2 IGRA Tube was coated with peptide components of the S1 domain of the SARS-CoV-2 spike protein; and (3) CoV-2 IGRA Stim was coated with mitogen to verify if the sample contained a sufficient number of viable and functional T cells. After incubation of the individual whole blood in the stimulation tubes for 20–24 h at 37 °C, the separated plasma was used to determine IFN-γ concentration by Quan-T-Cell ELISA. 

### 2.3. Humoral Immunity Analysis

Anti-SARS-CoV-2 QuantiVac ELISA IgG (Euroimmun Medizinische Labordiagnostika, Lübeck, Germany) was used for quantification of human antibodies of the immunoglobulin class IgG against the S1 domain of the SARS-CoV-2 spike protein in the sera of investigated individuals. Antibody titer was measured from the same blood sample to compare humoral and cellular immunity. 

All values below the cut-off value of 200 mIU/mL for IFN-γ concentration and 35.2 IU/mL for antibody concentration were reported as negative results [15]. 

### 2.4. Ethics Approval and Informed Consent

The ethics committee of St. Catherine Specialty Hospital approved this study. All participants provided written informed consent.

### 2.5. Statistical Analysis

Statistical analysis was performed in the software package IBM SPSS Statistics 23.0 (SPSS, Chicago, IL, USA), with a significance level of *p* < 0.05, and data were visualized using GraphPad Prism version 8.0.0 for Windows (GraphPad Software, San Diego, CA, USA). The normality of the distribution of individual parameters within the groups was tested using the Kolmogorov–Smirnov test of normality. Since the analysis showed a non-normal distribution of data, nonparametric statistical tests (Mann–Whitney test, Chi-square test, and Fisher’s exact test) were used.

## 3. Results

Participants were divided into different groups according to different infection status and vaccination history: Group A included participants with infection and with additional vaccination; Group B included participants with infection and without additional vaccination; Group C included participants without infection and with additional vaccination; Group D included participants without infection and without additional vaccination. Analyzed data obtained from the 303 participants are shown in Table 1. 

Statistical analysis showed no significant difference in the level of cellular immunity between 139 males (45.9%) and 164 females (54.1%) but showed higher antibody concentrations in males than females (Mann–Whitney, *p* = 0.015).

The Mann–Whitney test showed a significant difference in the level of cellular immunity between reinfected participants and those without infection after initial testing of IFN-γ (*p* = 0.012). A significantly higher level of cellular immunity was found in participants who were not infected or reinfected with the SARS-CoV-2 after vaccination and/or the previous SARS-CoV-2 infection. 

**Figure 1:**
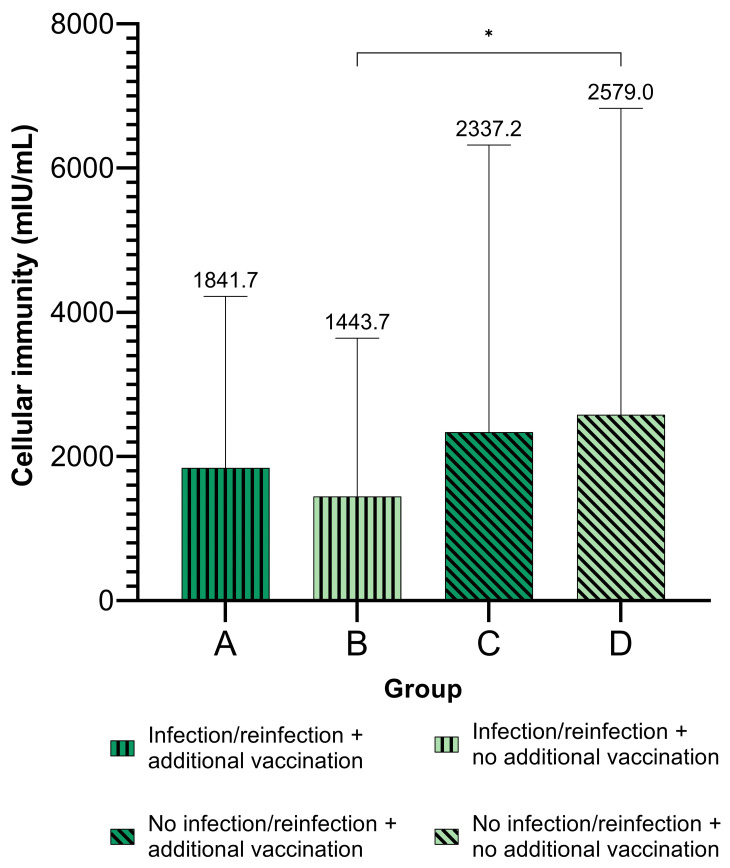
Level of cellular immunity (IFN-γ concentration) in patients with a SARS-CoV-2 infection or reinfection and additional vaccination after initial testing of IFN-γ. Group A—participants with (re)infection and additional vaccination; Group B—participants with (re)infection without additional vaccination; Group C—participants without (re)infection with additional vaccination; Group D—participants without (re)infection and additional vaccination. Mean values are shown above each bar. *—Mann–Whitney, *p* = 0.016.

Through further analysis, the participants were classified into two groups: those who received additional vaccination after measuring IFN-γ concentration (groups A and C) and those without additional vaccination (groups B and D) (Figure 1).

**Figure 2:**
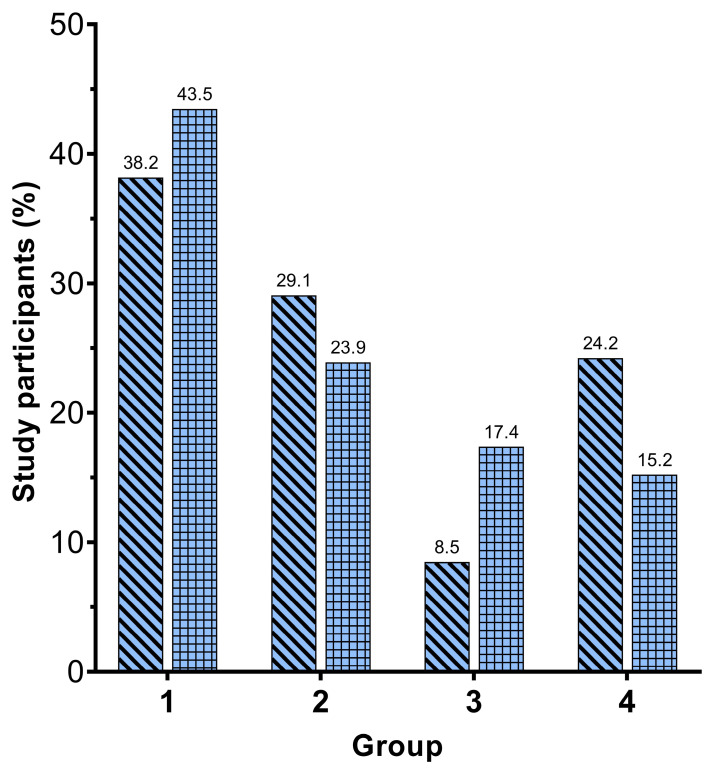
Percentage of study participants with and without a SARS-CoV-2 infection or reinfection (after IFN-γ test) in participants with previous COVID-19 (before IFN-γ test) (group 1), participants vaccinated with one of the SARS-CoV-2 vaccines (group 2), participants who had both the SARS-CoV-2 infection and vaccination history (group 3), and patients without a history of SARS-CoV-2 infection or vaccination (group 4). Groups 2 and 4 have a higher percentage of participants who were infected with SARS-CoV-2, while groups 1 and 3 have a higher percentage of participants who did not have a SARS-CoV-2 reinfection.

In study participants who received additional vaccine doses, the level of cellular immunity did not significantly differ between (re)infected and non-(re)infected group. On the other hand, in individuals without additional vaccination, those who experienced infection/reinfection (group B) had significantly lower levels of cellular immunity compared to the uninfected participants (group D) (Mann–Whitney, *p* = 0.016) (Figure 1). Moreover, participants with previous SARS-CoV-2 infection showed a reduced (re)infection/no (re)infection ratio compared with those who were only vaccinated before initial testing of IFN-γ (Figure 2). The Chi-square test showed a significant difference in (re)infection within different groups of participants, as shown in Figure 2 (*p* = 0.028).

## 4. Discussion

Our results showed that IFN-γ presumably plays an important role in the long-term cellular immune response and protection against SARS-CoV-2 variants of concern. A cohort study of 61 immunized subjects showed a persistent and robust T-cell response suggesting the extreme importance of cellular immunity in the fight against new variants and protection against severe COVID-19 [2,6]. The results of the study on macaques demonstrate that the cellular immune response is crucial in the case of suboptimal antibody titer levels [8]. 

Our results showed a significantly higher level of cellular immunity in individuals who were not infected or reinfected with the SARS-CoV-2 virus after vaccination and/or previous COVID-19 infection. Previous research established that cellular immunity does not wane and remains persistent up to 20 months after the last contact with the viral antigen in vaccinated participants and patients with a previous infection. Moreover, simultaneous measurement of antibody titers in the same subjects showed a significant decline in antibody titers after only six months [15]. These findings, along with the results which showed that patients who recovered from SARS possess long-lasting memory T cells that are reactive to the N protein of SARS-CoV 17 years after the outbreak of SARS in 2003, indicate how persistent and robust cellular immunity can be over a long period of time [16,17,18,19]. 

Furthermore, our research showed a reduced (re)infection/no (re)infection ratio in participants with previous SARS-CoV-2 infection compared with those who were only vaccinated. Groups of participants vaccinated with one of the SARS-CoV-2 vaccines and participants without a history of SARS-CoV-2 infection or vaccination had a higher percentage of participants who were infected with SARS-CoV-2, while groups of participants with previous COVID-19 (before IFN-γ testing) and participants who had both the SARS-CoV-2 infection and vaccination history have a higher percentage of participants who did not have a SARS-CoV-2 reinfection. The results of our research, combined with the findings of other studies, indicate that IFN-γ presumably plays an important role not only in T-cell activation and early protective response to COVID-19 disease but also in preventing SARS-CoV-2 infection and SARS-CoV-2 reinfection after vaccination [20,21]. We therefore propose that there is a reduced level of cellular immunity in participants with a SARS-CoV-2 infection or reinfection as measured by the IFN-γ concentration. The concentration of IFN-γ was presumably associated with lower cellular immune protection, including lower activation and induction of macrophage production, and consequently a higher rate of infection/reinfection.

Our findings suggest a long-lasting effect of cellular immunity (measured by IFN-γ concentration), which through precise modulation plays a key role in preventing infections and reinfections during the current pandemic and has implications for the development of SARS-CoV-2 vaccines and immune-based therapeutic agents based on the cellular immune response.

## Figures and Tables

**Table 1:** Baseline Demographics and Cohort Characteristics (N = 303). Group 1: Infected/reinfected participants; Group 2: Not infected/reinfected participants. MD, median; IQR, interquartile range; M, male; F, female. **— *p* < 0.001 (Fisher’s exact test); *— *p* < 0.05 (Mann–Whitney test).

		Group 1 (N = 165)	Group 2 (N = 138)	*p* Value (Group 1 vs. Group 2)
Cellular immunity (mIU/mL) (N = 303)	MD	768.0	958.5	0.012 *
	IQR	1906.1	2553.8	
Antibodies (IU/mL) (N = 303)	MD	120.9	156.9	0.010 *
	IQR	369.2	747.8	
Age (years) (N = 303)	MD	50.0	51.0	0.127
	IQR	18.0	19.25	
Sex, No. (N = 303)	M	72/165 (43.6%)	67/138 (48.6%)	0.419
	F	93/165 (53.4%)	71/138 (51.4%)	
No symptoms during (re)infection		26/165 (15.8%)	/	/
Mild symptoms during (re)infection		134/165 (81.2%)	/	/
Severe symptoms during (re)infection		5/165 (3.0%)	/	/
SARS-CoV-2-positive family members		141/165 (85.5%)	86/138 (62.3%)	<0.001 **
Negative family history of SARS-CoV-2		24/165 (14.5%)	52/138 (37.7%)	
Vaccinated participants (before the IFN-γ test)		62/165 (37.6%)	57/138 (41.3%)	/
Infected participants (before the IFN-γ test)		77/165 (46.7%)	84/138 (60.9%)	/
Additionally vaccinated (after the IFN-γ test)		34/165 (20.6%)	41/138 (29.7%)	/

## Data Availability

The data sets generated during this study are available from the corresponding author on request.

## Limitations of the Study

Study limitations include a small sample size resulting in greater data variability, absence of a group with no vaccination and no infection, and measurement of only IFN-γ concentration, without investigation of other immune response components. Participants received different vaccine types (Pfizer, Moderna, AstraZeneca, and Johnson & Johnson), potentially impacting the cellular immune response. Specific variant identification was not performed, although assumed to be Delta and Omicron during the study period. No sex differences were found in cellular immunity, possibly due to the small sample size. Limitations must be considered when interpreting results, and larger studies may be necessary to confirm findings.
